# *Salmonella* Senftenberg Infections and Fennel Seed Tea, Serbia

**DOI:** 10.3201/eid1605.091555

**Published:** 2010-05

**Authors:** Svetlana Ilić, Predrag Đurić, Edita Grego

**Affiliations:** Institute of Public Health of Vojvodina, Novi Sad, Serbia (S. Ilić, P. Đurić); Institute of Public Health of Serbia, Belgrade, Serbia (E. Grego)

**Keywords:** Outbreaks, epidemiology, Salmonella Senftenberg, Foeniculum, bacteria, letter, *Suggested citation for this article*: Ilić S, Đurić P, Grego E. *Salmonella* Senftenberg infections and fennel seed tea, Serbia [letter]. Emerg Infect Dis [serial on the Internet]. 2010 May [date cited]. Available from http://www.cdc.gov/EID/content/16/5/893.htm

**To the Editor:** The first documented outbreak of salmonellosis linked to consumption of plant products in the Autonomous Province of Vojvodina, Serbia, occurred from March 2007 through September 2008. Fourteen cases of *Salmonella* *enterica* serotype Senftenberg infection were reported.

The yearly incidence of salmonellosis in Vojvodina during 2003–2007 ranged from 25/100,000 inhabitants to 70/100,000 inhabitants; 34 outbreaks were reported in 2007, caused predominately by *S.* *enterica* serotype Enteritidis (*1*). Most outbreaks were associated with consumption of food of animal origin (*1**,**2*). *Salmonella* spp. were isolated from seeds in 2004, when *S.* *enterica* serotype Mbandaka and *S.* *enterica* serotype Virchow were isolated from sesame seeds (*3*).

Before 2007, *S.* Senftenberg had rarely been identified in Vojvodina. During 2003, 3 cases were reported. In 2004–2005, no *S*. Senftenberg cases were reported. In 2006, 8 cases of *S.* Senftenberg infection were reported among infants <12 months of age. An outbreak investigation did not reveal the source of infection. Common to all of those infected was their age and their consumption of infant formula. Nonetheless, laboratory analysis of samples of the various formulas did not show any pathogens. Two additional cases occurred in 2007 among patients who were <12 months of age. These cases confirmed suspicion that the infections had a source other than formula. Further investigation led to the consideration of tea consumption as a possible factor.

In April 2008, a total of 3 infants <12 months of age with salmonellosis came to the attention of investigators. *S.* Enteritidis was first identified in the samples of their feces. One month later, feces samples from the 3 infants were tested again, and *S.* Senftenberg was isolated from all 3 specimens.

After these findings, the Institute of Public Health of Vojvodina conducted an outbreak investigation in collaboration with institutes of public health at the district level. A case was defined as the presence of a laboratory-confirmed *S.* Senftenberg infection during 2007–2008. All case-patients (or their parents) were interviewed by using a standard questionnaire for salmonellosis, which was expanded to include questions regarding tea consumption.

A standardized method of enterobacterial repetitive intragenic consensus (ERIC)–PCR, based on the method of Versalovic et al. (*4*), with ERIC-PCR with ERIC2 primer (5′AAGTAAGTGACTCGGGTGAGCG-3′), was applied. DNA was isolated by using the InvitrogenPure Link Genomic DNA purification kit (Invitrogen, Carlsbad, CA, USA). Gene sequences were amplified in a Perkin/Elmer thermal cycler (model 9600) (PerkinElmer, Waltham, MA, USA). A DNA ladder was constructed by using Gene Ruler 100-bp DNA Ladder Plus (Fermentas, Glen Burnie, MD, USA).

Exploratory interviews with parents showed that all 3 infected infants had consumed commercially manufactured baby tea during the previous month (after diagnosis of *S.* Enteritidis infection was made). Before feeding it to the infants, the parents had not heated the tea until it boiled, but rather had poured boiled water over the tea. After obtaining that information, we tested 33 samples of the incriminated brand of tea from public grocery stores and supermarkets; 13 samples were positive for *S.* Senftenberg. The organism’s genetic profile was identical or similar from both tea and human samples (Figure).

**Figure:**
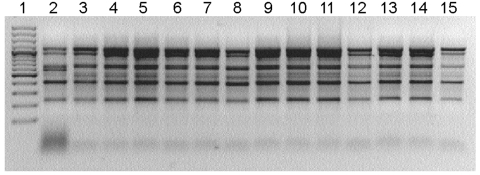
Enterobacterial repetitive intragenic consensus (ERIC)–PCR ERIC2 primers. Lane 1, molecular mass ladder; lanes 2–7, nonoutbreak isolates; lanes 8–9, isolates from baby tea; lane 10, isolate from fennel; lanes 11–15, isolates from salmonellosis patients. ERIC PCR with ERIC2 primer (5′-AAGTAAGTGACTCGGGTGAGCG-3′) was used. DNA was isolated by using the InvitrogenPure Link Genomic DNA purification kit (Invitrogen, Carlsbad, CA, USA). Gene sequences were amplified in a Perkin/Elmer thermal cycler (model 9600; PerkinElmer, Waltham, MA, USA). A DNA ladder was created by using Gene Ruler 100-bp DNA Ladder Plus (Fermentas, Glen Burnie, MD, USA).

Baby tea, widely distributed throughout Serbia, contains aniseed and caraway and fennel seeds. Sanitation inspectors collected samples from tea manufacturers. In the fennel seed sample, *S.* Senftenberg was identified. According to the tea manufacturer, fennel was purchased from another company, which collected seeds from individual producers. Fennel seed was cultivated in a household garden by an unregistered producer; neither the grower nor fennel stocks could be found. Two cases of *S.* Senftenberg from 2007 were retrospectively linked to infant tea, as were all other cases reported in 2008.

Demographic characteristics and clinical status of the case-patients were analyzed. Of 14 cases of *S.* Senftenberg infection, 10 were in infants <12 months of age (average 5.1 months). Half had diarrhea and the same proportion had fever >38.5˚C. Ten patients were female and 4 were male. All 4 adults had mild infection, except 1 adult who had concomitant *Clostridium* *difficile* infection. Three infants and an adult with concomitant infection were hospitalized.

Most infections were reported in May 2008, including the 3 cases in infants who were recovering from *S.* Enteritidis infection. After September 2008, no new cases of *S.* Senftenberg were reported until July 2009, when 1 case was identified in a 24-year-old man.

The heat resistance of *S.* Senftenberg is well known and is much higher than that for most other *Salmonella* serotypes (*5*). A number of recent outbreaks of *S.* Senftenberg infection resulted from consumption of fresh products. Thus, products that will be used in a fresh state should undergo more rigorous testing for pathogens, or better methods of infection control must be used.

The European Food Safety Authority has noted that all botanicals or botanical preparations could become hazardous as a result of flaws in the production process; therefore, manufacturers should follow the Hazard Analysis and Critical Control Point systematic approach (*6*). This system must be applied with the necessary flexibility and adapted to each botanical preparation on a case-by-case basis.

In 1999, the US Food and Drug Administration recommended that seeds be disinfected by washing with calcium hypochlorite solution before they sprout. However, this treatment destroys only pathogenic microorganisms on the seed surface (*7*,*8*). Thus, new methods, such as high hydrostatic pressure or use of bacteriophages as biocontrol agents should be adopted. High-pressure processing does not change the taste of food or cause any physical damage (*7*). With further refinement of phage delivery mechanisms, *Salmonella* phages could be effective in eliminating or reducing *Salmonella* contamination of vegetables (*9*).
